# Effects of stimulus polarity on the local evoked potential in auditory brainstem implant users

**DOI:** 10.1038/s41598-025-90114-z

**Published:** 2025-02-18

**Authors:** Anne Schröder, Marko Takanen, Konrad Schwarz, Thomas Lenarz, Lutz Gärtner, Andreas Büchner

**Affiliations:** 1 MED-EL Deutschland GmbH, Starnberg, Germany; 2 Department of Otolaryngology, Hannover Medical School, Carl-Neuberg-Straße 1, 30625 Hannover, Germany; 3 MED-EL GmbH, Innsbruck, Austria; 4 Cluster of Excellence “Hearing4All”, Hannover, Germany

**Keywords:** Auditory brainstem implant, Neural prosthesis, Cochlear nucleus, Local evoked potential, Auditory perception, Audiology, Cochlea, Cortex, Inner ear, Biomedical engineering, Cancer in the nervous system, Developmental disorders

## Abstract

Auditory brainstem implants (ABI) can enable hearing sensation through electrical stimulation of the cochlear nucleus. The basic stimulation and signal coding strategies of the ABI are based on those of the cochlear implant. This may not always be optimal, and ABI-specific strategies may be preferred. In a cohort of ten ABI users, we examined the feasibility of measuring local evoked potentials (LEP) via fine-grained stimulation with a forward masking paradigm. We introduce a new baseline-dependent definition of LEP amplitude for analyzing the LEP amplitude growth function to obtain threshold stimulation levels and slope values. The processing of biphasic pulses by the cochlear nucleus and the influence of the leading phase polarity were examined. There were no statistically significant differences in LEP thresholds or slopes between cathodic and anodic leading pulses. LEP thresholds measured with cathodic leading pulses (r = 0.77, *t*_31_ = 6.81, *p* < 0.0001) and anodic leading pulses (r = 0.70, *t*_27_ = 45.14, *p* < 0.0001) correlated significantly with perceptual hearing thresholds. The correlation analysis was impacted by outlier values, especially in the case of LEP thresholds measured with anodic leading pulses. Cathodic leading pulses had significantly shorter LEP peak latencies (*t*_104.8_ = 2.63, *p* < 0.01). These results show that the cathodic leading pulses are superior for eliciting LEPs. We suggest that cathodic leading pulses should be the basis for ABI-specific coding strategies.

## Introduction

Cochlear implants (CI) are the standard of care for individuals with severe to profound hearing loss. Cumulatively, more than one million devices have been implanted^1^. Over the decades, there have been many improvements in the areas of hardware design, sound coding strategies, and in surgical and fitting procedures for CIs^2^. One productive area of research has been in the development of objective measures which can assess both the functionality of the device and the electrode neuron-interface. These measures can also assist in optimizing fitting parameters. This may be particularly useful with individuals for whom behavioral testing can produce less reliable results, such as young children, those with intellectual or cognitive comorbidities, and other individuals who cannot provide reliable reports of their hearing percepts^3^.

The CI audio processor splits the audio signal into separate channels via bandpass filters and converts the signal at each channel into an electrical pulse train which is delivered to an array of intracochlear electrodes^4^. These pulse trains are typically biphasic and are charge-balanced in order to protect the cochlea from charge buildup^5,6^. The neural signals evoked by the electrical stimulation traverse along the cochlear nerve to the cochlear nucleus (CN) of the auditory brainstem, which processes the input and projects responses to different targets along the auditory pathway^7^.

A CI therefore requires both an intact cochlea and an intact cochlear nerve. This is not always the case, and this led to the development of a post-cochlear nerve auditory prosthesis—the auditory brainstem implant (ABI). The ABI is indicated as an alternative treatment option if no benefit from a CI is to be expected^8^. The pathologies for which the ABI is indicated include the presence of non-functioning cochlear nerves after tumor resection in neurofibromatosis type 2 (NF2), congenital malformations of the inner ear which preclude cochlear implantation, and cochlear nerve hypoplasia or aplasia^9^.

The first ABI was implanted by Hitselberger in 1979^10^. The general hardware design and signal processing strategies of the ABI have been—then and now—based on that of the CI. There are many dissimilarities between the neural tissues and environments to which the electrical stimulation is applied with an ABI versus a CI. In contrast to the cochlea, the CN is not tonotopically organized on the outer layer, where the ABI electrode panel is placed^11^. The CN also contains neural structures that do not process auditory signals, and stimulation of these structures by the ABI can produce non-auditory side effects such as coughing, tingling or dizziness^12^. As a result of these difference, there may be scope for improvement in ABI performance through the development of ABI-specific signal processing strategies.

**Fig. 1:**
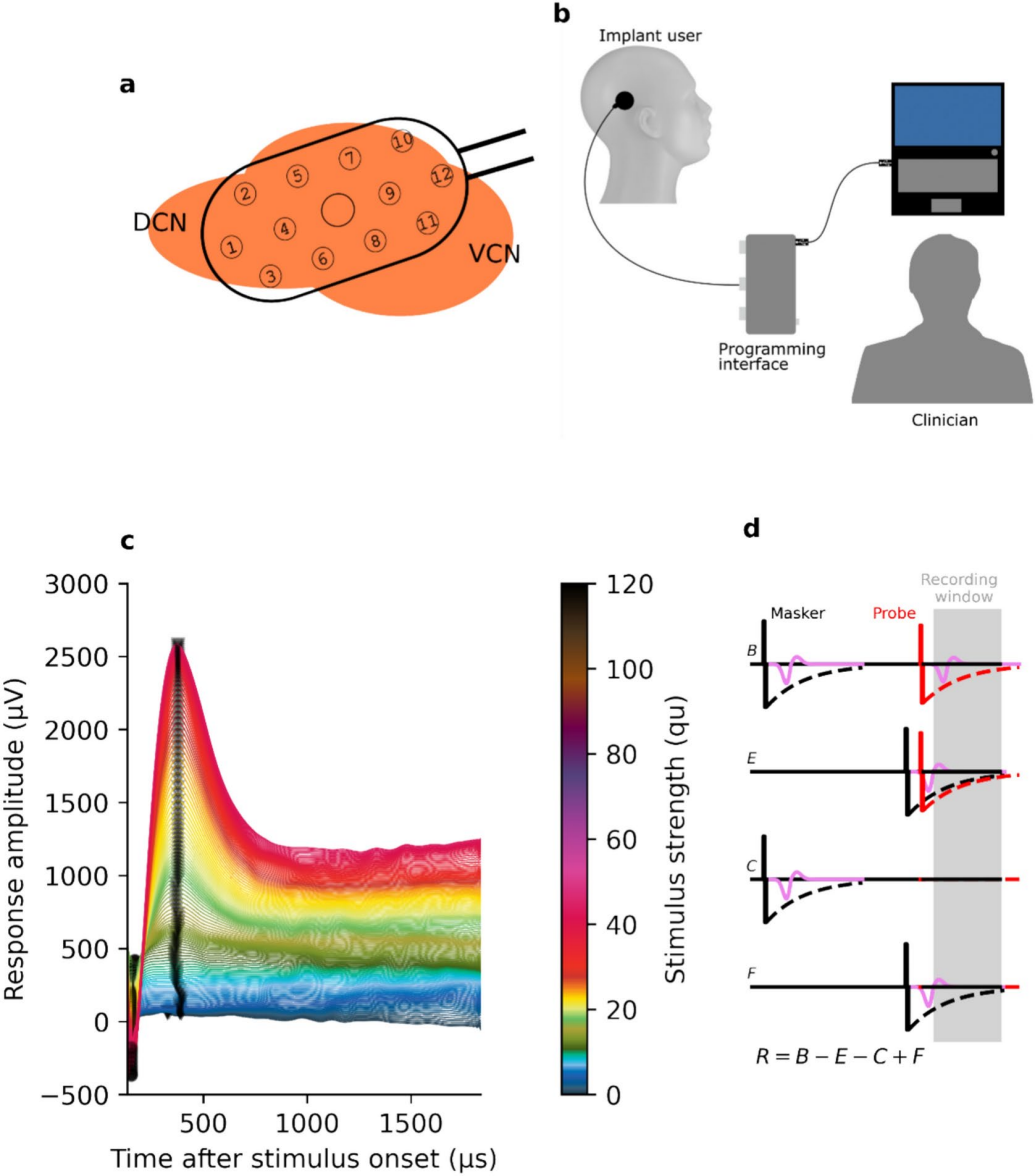
(**a**) Schematic illustration of a typical position of a ABI electrode panel on the ventral (VCN) and dorsal cochlear nucleus (DCN). (**b**) Illustration of the stimulation and recording setup. The implant was connected via a transmitting coil to the programming interface, which was controlled by a computer. (**c**) Plot of exemplary LEP responses for one participant measured at a single recording electrode. To ease visual inspection, vertical offset has been introduced between traces, and stimulus intensities have been color coded. (**d**) Pulse sequence for measuring LEPs. The strengths of the masker and the probe pulses were incrementally increased according to the fine-grain stimulation paradigm. The neural responses elicited by the probe and masker pulses are depicted as purple signals and the dashed black and red lines depict stimulation artefacts related to the masker and probe pulse, respectively.

To achieve this, a better understanding of the responses of CN neural structures to ABI stimulation is needed. Biphasic pulses are known to reduce the risk of tissue damage caused by charge buildup^5,13^, which is why they are the standard for both the CI and ABI. However, a challenge which arises when stimulating the CN compared to stimulating the cochlea is that it is comprised of a complex and heterogeneous population of neural cell types^14^. The electrode paddle is placed onto both the dorsal and ventral surfaces of the CN, as depicted in Fig. 1a. The dorsal CN contains excitatory neurons including fusiform and giant cells which are responsible for signal transduction, while tuberculoventral and cartwheel cells are inhibitory and do not project to other structures. The ventral CN consists of cell types such as bushy and stellate cells which project to the superior olivary complex and the inferior colliculus^14,15^.

For both the CI and the ABI, electrical stimulation generates an action potential in the neurons proximal to the electrode. The strength of the electrical stimulus is usually correlated with the perceived loudness sensation. The summation of the activity of excited nerve fibers yields an electrically evoked compound action potential (eCAP). This response can be recorded at a different electrode contact using two-way telemetry. In the CI, the eCAP is well understood and provides a valuable tool for testing the integrity of the electrode-nerve interface. eCAP measurements have thus found their way into clinical practice, for both intraoperative and postoperative routines^16^.

**Fig. 2:**
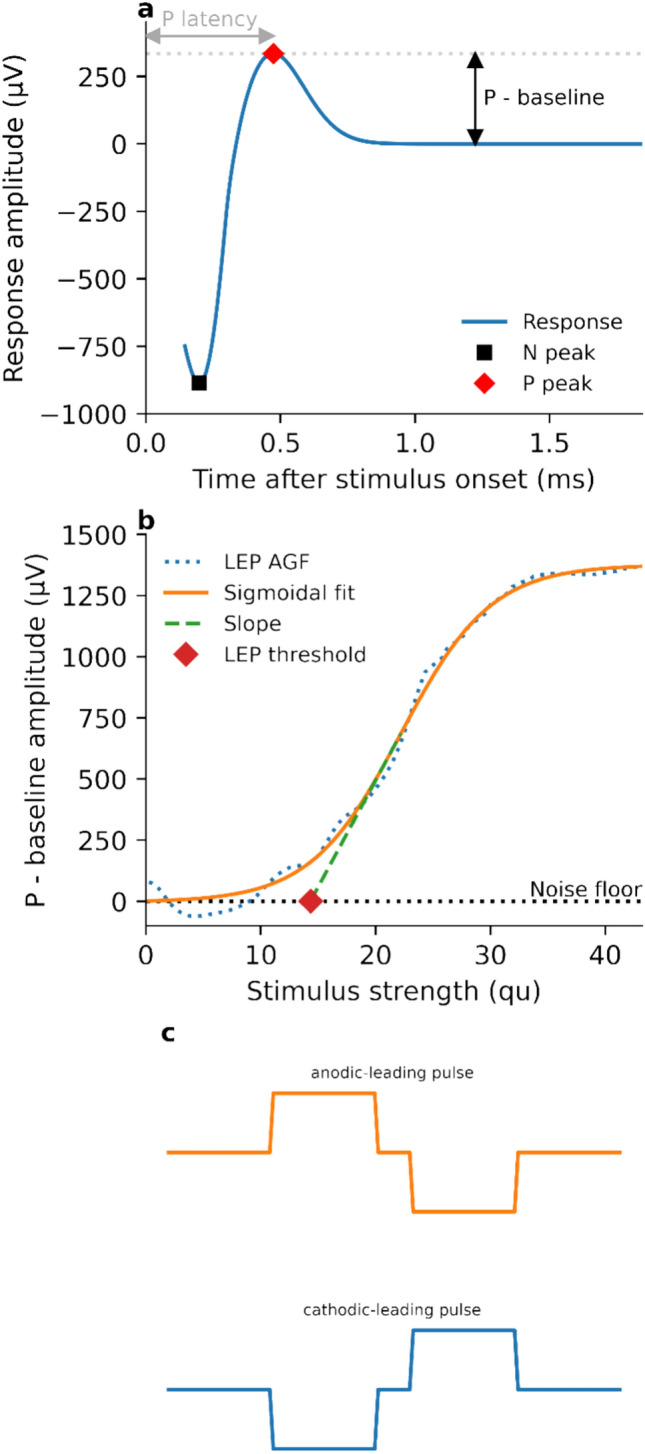
(**a**) Schematic illustration of LEP responses. The baseline, N and P peaks, and P latency are indicated. (**b**) A LEP amplitude growth function. The dotted line denotes measured LEP amplitudes, and the solid line denotes the sigmoidal function fitted to the measured values. The threshold value and slope (dashed line) of the fitted sigmoid function are indicated. (**c**) Illustration of the anodic- and cathodic-leading biphasic charge-balanced pulses used for stimulation. The zero-amplitude portion between the positive and negative phases is denoted as inter-phase gap.

In the CN, the equivalent of the eCAP is referred to as the local evoked potential (LEP). As described by Gärtner et al.^17^, LEPs show a very similar morphology to eCAPs, with a characteristic negative-going peak followed by a positive-going peak^18^ (illustrated in Fig. 2a). The voltage difference of the minimum (N) and maximum (P) peak amplitudes is defined as the LEP amplitude. By plotting LEP amplitude as a function of stimulation level, an amplitude-growth function (AGF) is obtained. For clinical practice, the AGF parameters of slope and threshold stimulation level (illustrated in Fig. 2b) are of interest, as these can be used to estimate the integrity of the electrode-neuron interface and can be indicative of perceptual thresholds, respectively^17^.

In audiological fitting sessions, perceptual thresholds are typically determined behaviourally by stimulating single electrodes while the user is asked about their auditory impression. The lowest stimulus strength where the user reports a sound sensation is set to be the perceptual hearing threshold. However, obtaining this feedback from user can be challenging for several reasons, for example a lack of experience with sound sensation, mental comorbidities or in pediatric users that cannot give reliable verbal feedback of their sensations. The use of objective measures such as the LEP may enable the estimation of perceptual thresholds in these cases.

Several challenges do remain, which hinder the prospect of using LEPs as objective measures of hearing. Not all electrode channels will produce a hearing sensation. It is a difficult task to insert the electrode array into the lateral recess and onto the CN. It is not clear whether the electrode array will be placed over excitable neurons. Due to the variety of cell types in the CN and in structures immediately adjacent to it, which may also be subject to electrical stimulation, sometimes there is hearing percept, sometimes there is not, and sometimes a non-auditory percept is evoked. Nevison et al.^19^ observed that side-effects due to stimulation of non-auditory neurons occur in almost all subjects—only 7.7% of individuals reported complete absence of non-auditory sensations.

In contrast to eCAPs, the LEP N peak is often cut-off in the recording. This is due to the presence of the electrical stimulus artefact, which is also picked up by the recording electrode. The stimulus artefact is typically orders of magnitude larger than the neural response (volts versus microvolts). As the response needs to be amplified prior to transmission, the recording system must wait until the residual charge of the stimulus artefact has decayed to a level that it will not cause saturation of the amplifier, which would induce clipping of the response. In the eCAP, the neural structures being stimulated lie a considerable distance from the electrode array. As such, the latency between the stimulus and response is typically large enough that there is little or no temporal overlap between them. In contrast, the ABI electrodes are placed directly on surface of the CN, and the latency between stimulus and response is correspondingly shorter. For this reason, an alternative method for analyzing and interpreting LEP responses is needed which does not rely on the N peak.

LEPs have been studied in ABI users by performing several repetitive measurements on discrete stimulus strengths^17^. It is also possible to use the fine-grain paradigm to sample the eCAP/LEP amplitude growth function in finer detail by increasing the stimulus strength in small increments and recording eCAPs/LEPs at each increment. As this produces a single response for each stimulus strength, no averaging of responses is used^20^. Gärtner et al.^17^ introduced the measuring of LEP and showed a significant correlation between LEPs and hearing perception. In 90.5% of the cases where LEPs could be detected there was also a reported hearing percept. However, that study also reported 33.7% false positive results.

The present study was performed with a cohort of ten ABI users, and had three broad objectives. The first was to determine the feasibility of using the fine-grain stimulation paradigm for measuring LEPs in ABI users. Part of this objective was to determine a method of estimating LEP amplitudes when the N peak is absent from the processed recording which, as mentioned above, is a common situation. To that end, we introduce a new definition of LEP amplitude which is independent of the N peak.

The second objective was related to the wider goal of developing an ABI-specific signal coding strategy. Here, we studied the influence of biphasic pulse polarity (cathodic-leading, CA, versus anodic-leading, AC) on the excitability of CN neural structures as measured with LEPs. Differences between the two polarity configurations were hypothesized to manifest as alterations in LEP latencies and/or in the thresholds or slopes of the LEP AGF. To facilitate investigation of polarity-related effects, LEPs were recorded using the revised forward-masking paradigm^21^ for artefact reduction.

The third objective was to determine the feasibility of using LEPs as a supplement to behavioural testing for fitting in ABI users. We investigated the relationship between the stimulation thresholds measured from LEP AGFs and the perceptual thresholds obtained from clinical fitting session of the ABI users. We hypothesized that these two threshold measures would correlate sufficiently to allow LEP thresholds to be used to predict perceptual thresholds. These correlation studies were performed separately for both cathodic-leading and anodic-leading pulse shapes, in alignment with the second objective.

## Results

### Participants

In total, ten ABI users were enrolled. Six were young children aged between 3 and 7 years of age and four were adults. The duration of ABI experience (interval between implantation and measurement) ranged from less than one year to 11 years (median 6 years). More details of demographic and device characteristics are provided in Table 1.

**Table 1:** Demographic data of the participants and implant characteristics.

ID	Implant	Side of implantation	Age at implantation (years)	Age at measurement (years)	Etiology
01	SYNCHRONY ABI	Right	34	34	Neurofibromatosis Type II
02	PULSAR ti100 ABI	Right	47	58	Meningitis
04	SYNCHRONY ABI	Right	4	8	Meningitis
05	CONCERTO ABI	Right	7	15	CHARGE syndrome/cochlear dysplasia
06	SYNCHRONY ABI	Left	4	9	CHARGE syndrome/cochlear dysplasia
07	CONCERTO ABI	Right	61	69	Cochlear otosclerosis
09	SYNCHRONY ABI	Right	4	12	Goldenhar syndrome
10	SYNCHRONY ABI	Left	60	62	Meningitis
16	SYNCHRONY ABI	Right	3	8	Cochlear nerve aplasia
17	CONCERTO ABI	Right	3	12	Cochlear aplasia

Age was rounded to whole years.

### Morphology of LEP responses

In total 60,491 individual LEP responses were acquired in the study. On average, approximately 444.8 individual responses (SD: 180) were acquired for each participant at each stimulating electrode for both pulse polarities. The number of electrode contacts at which the responses were recorded for each participant are given in the supplementary data. LEP responses were examined by plotting the response amplitude as a function of time after stimulus onset. LEPs were characterized by an early negative-going peak followed by a steep positive-going peak, with the peak maxima denoted here as N and P, respectively (Fig. 2a).

When the LEP response amplitude (P—baseline) was plotted as a function of the stimulus strength, a generally monotonic and sigmoidal AGF was observed (Fig. 2b). The slopes and thresholds of these AGFs were determined as described in the methods.

### Effect of pulse polarity on LEP thresholds and the slope of the LEP AGF

**Fig. 3:**
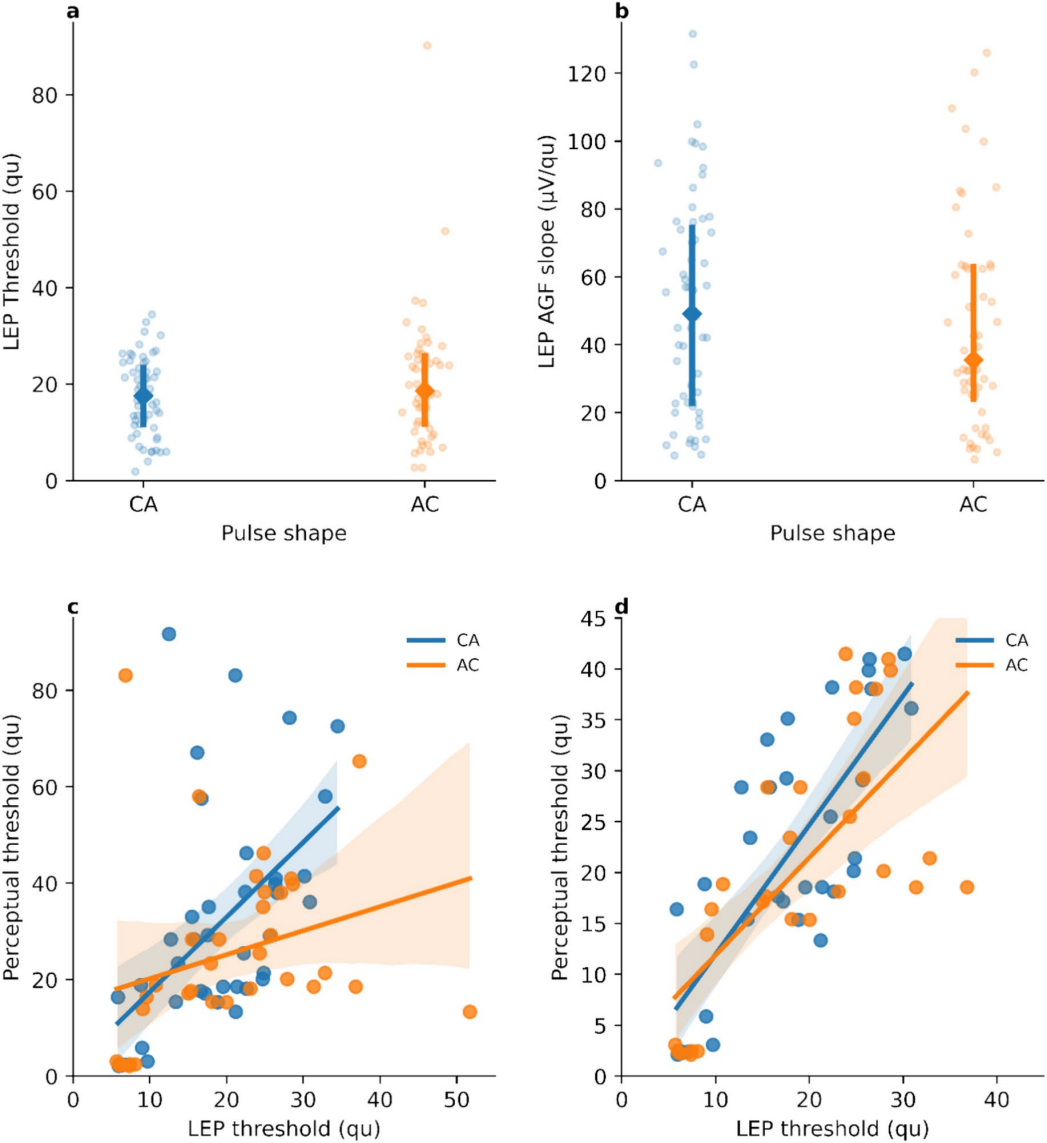
Distributions of (**a**) LEP thresholds and (**b**) LEP AGF slopes for the two pulse polarities CA and AC. The median values and inter-quartile ranges are indicated. Relationships between LEP thresholds and perceptual thresholds for (**c**) the complete data and (**d**) upon exclusion of outlier values (LEP and perceptual thresholds above 45 qu), respectively. Regression lines are shown. The error bands on the regression lines represent the 95% confidence intervals of the regression.

ANOVA of linear mixed effects models yielded non-significant results for all dependent variables (polarity, electrode and their interaction), indicating that there were no statistically significant differences between anodic- and cathodic-leading pulses on LEP thresholds or on LEP AGF slopes. The median values and the quartile ranges of the LEP thresholds and the AGF slopes are shown in Fig. 3a and b, respectively.

### Correlation between pulse polarity and subjective hearing thresholds

LEP thresholds stimulated from cathodic-leading pulses were found to correlate strongly with perceptual thresholds (r = 0.54, *t*_39_ = 4.00, *p* < 0.001), while those stimulated from anodic-leading pulses did not significantly correlate with perceptual thresholds (r = 0.29, *t*_32_ = 1.70, *p* > 0.05) (Fig. 3c).

The correlation analysis was found to be affected by outlier threshold values above 45 qu (charge unites, approximately equivalent to 1 nC). These values were beyond 1.5 times the inter-quartile ranges for LEP and perceptual thresholds, and may result from the electrodes stimulating primarily (imperceptible) non-auditory activity. Upon exclusion of these outliers, perceptual thresholds were found to correlate strongly with LEP thresholds measured with both cathodic- and anodic-leading pulses (r = 0.77, *t*_31_ = 6.81, *p* < 0.0001 and r = 0.70, *t*_27_ = 45.14, *p* < 0.0001, respectively) (Fig. 3d).

### Effect of pulse polarity on P latencies

The mean P latencies were shorter for cathodic-leading pulses (462 µs ± 109 µs SD) than for anodic-leading pulses (521 µs ± 126 µs SD). Paired t-tests indicated that these differences were statistically significant (*t*_104.8_ = 2.63, *p* < 0.01).

**Fig. 4:**
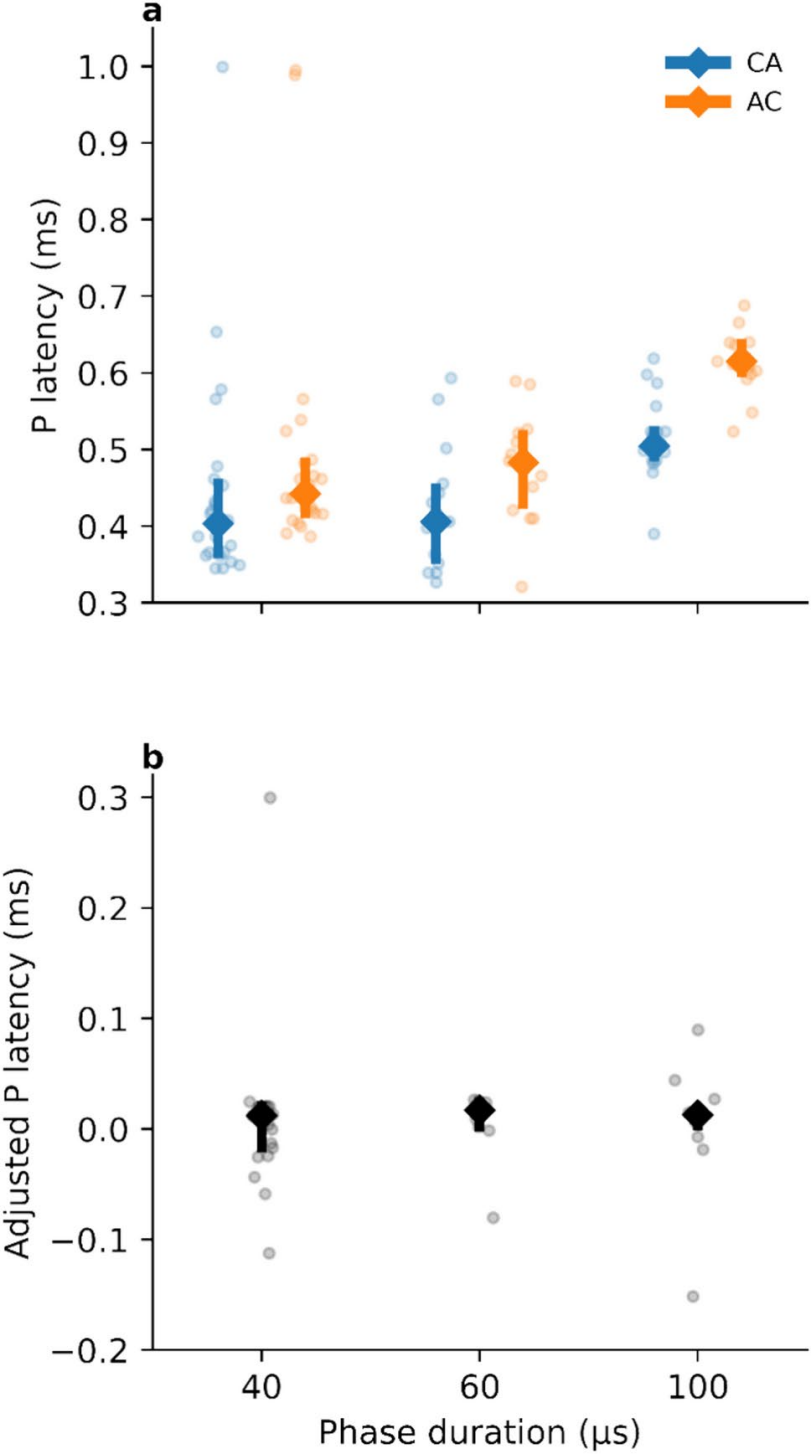
(**a**) P latencies as a function of phase duration. Shorter P latencies were observed for cathodic-leading pulses with all phase durations. (**b**) Adjusted P latencies as a function of phase duration. Latencies were adjusted by subtracting the latencies for CA pulses, the inter-phase gaps and the phase durations from the latencies for AC pulses.

As stated in the methods section, the phase duration was automatically adjusted by the research software for each participant to ensure that the compliance limit was not exceeded. Therefore, the P latencies were also investigated as a function of phase duration. Figure 4a shows the distributions of P latencies for cathodic- and anodic-leading pulses as a function of phase duration. It was observed that as phase duration increased, the differences in P latencies between cathodic- and anodic-leading pulses increased. This finding suggests that CN neurons are more sensitive to excitation by cathodic-leading pulses. To test this idea, adjusted latency differences were computed by subtracting (separately for each electrode and each subject) the latency for the cathodic-leading pulse, the inter-phase gap (IPG, 2.1 µs) and the phase duration from the latency for the anodic-leading pulse. The P latencies for anodic-leading pulses were similar to those for cathodic-leading pulses when the phase duration and the IPG were compensated for (Fig. 4b). ANOVA showed that the adjusted latency difference was not significantly different from zero (*t*_39_ = 0.66, *p* > 0.5) and that this does not depend on the phase duration (F_2,39_ = 0.03, *p* > 0.9). This suggests that for anodic-leading pulses, the onset of the LEP response is associated with the second (cathodic) pulse. In other words, a response is not initiated until a cathodic pulse is presented. This suggests that the cathodic phase is largely or entirely responsible for eliciting the LEP.

## Discussion

In this study, electrically evoked LEPs were stimulated and recorded in ABI users. Symmetric charge-balanced biphasic pulses with different leading polarities were used, along with a revised forward-masking paradigm for artefact reduction. From these responses, the LEP AGF thresholds and slopes as well as P latencies were estimated. It was observed that P latencies were significantly shorter when cathodic-leading pulses were used. Both cathodic- and anodic-leading pulses elicited LEPs with similar thresholds and slopes. LEP thresholds were also compared with perceptual threshold data from routine audiological fitting, and a significant positive correlation was observed between electrophysiological thresholds and perceptual thresholds. The power of the correlation analysis was affected by outlier values, especially in the case of LEP thresholds measured with anodic-leading pulses. Taken together, these findings suggest that cathodic-leading pulses are superior to anodic-leading pulses for eliciting LEPs in ABI users and may also have clinical utility as an objective measure to estimate perceptual thresholds in users for whom behavioural testing is difficult or not possible.

The morphology of the LEP responses measured here was similar to that of the eCAP elicited via the CI^17^. The forward-masking method used in this study did not alter the characteristic morphology of the response, which is associated with a distinct maximum positive amplitude (P)^18^. Conventionally, a signal blanking technique is used for stimulus artifact reduction. However with the LEP response, the N peak can appear with a latency short enough that it occurs within the blanking timeframe itself and is thus removed, or it can be obscured by the stimulus artifact^22^. This occurs so frequently in measurements with the ABI that it introduces a systematic error in calculating the LEP amplitude from the difference between N and P. Therefore, a new definition of LEP amplitude relative to the baseline was used here and shown to be feasible. A consequence of this new definition is that care must be taken with comparing absolute values of the AGF slope, as the slope is presumably significantly altered by this definition. The AGF threshold depends solely on the onset of P and is therefore not affected.

The excitability of the CN cell bodies was found to depend on the leading polarity of the electrical stimulus. However, we found that LEP threshold levels and the slopes of the LEP AGFs were not significantly different between polarities. This finding diverges from those of other studies when investigating these parameters with CIs in animal models and human users^23–25^.

The polarity of the pulse was also found to significantly affect P latency. This observation supports the idea that the CN cell bodies are sensitive only to cathodic (i.e., negative) polarity pulses. This is in agreement with results from extracellular in vivo recordings on cat spinal motor neurons^26^. That CN cell bodies are sensitive to cathodic polarity may also explain why neither the LEP thresholds nor the slopes of the LEP AGF were found to depend on the pulse polarity. Starting with an anodic phase may build up a potential that must then be overcome by the following cathodic phase. Anodic-leading stimulation could, therefore, increase the threshold, and lower the maximum of the LEPs, which is partially reflected in the non-significant difference in the LEP AGF slopes shown in Fig. 3b. This contrasts with the CI, where several studies have shown that anodic-leading pulses more effective^27–29^ and are associated with a lower degree of facial nerve stimulation^30,31^.

As stated in the introduction, the CN contains a variety of different cell types with different functions^5,13–15,32^. Consequently, not only does the ABI stimulate a complex neural environment, but different electrodes on the ABI may face regions of the CN with entirely different cellular populations. This makes it difficult to determine the exact origin and nature of the responses to the different pulse polarities observed here. Furthermore, this may also explain the discrepancy of the latency differences not being reflected in threshold values, as is observed in single-fibre recordings and compound responses obtained with CIs. In electrical stimulation with the CI, the latency difference has been suggested to stem from different parts of the spiral ganglion nucleus being sensitive to the different pulse polarities, wherein an action potential initiated at peripheral processes would have longer latency because the signal would have to traverse the cell body^25^.

LEPs are likely correlated with hearing sensation, perceptual thresholds, and most comfortable loudness levels (MCLs) when using alternating polarity as an artifact reduction method^17^. In this method, the responses obtained with anodic- and cathodic-leading pulses are averaged, and a zero-amplitude template is subtracted. Our observations suggest that this might not be the method of choice, as there was a significant difference in P latency between the two polarity configurations. If this is generally true, then averaging over the cathodic and anodic polarities is likely to lead to peak smearing. Instead, the present work would suggest using an artifact reduction method based on recordings using a single pulse polarity, either based on templates or based on masking phenomena^33^.

In our measurements, the LEPs show a medium to strong correlation with perceptual thresholds. As mentioned before, obtaining precise feedback from the user about their loudness perception can be challenging. Because of this, further analysis of the MCL was not performed.

Electrophysiology studies of ABI users can be challenging for two main reasons. Firstly, the rarity of this indication means that at any given clinic, the available cohort size is usually very limited. Secondly, the etiologies for which an ABI is indicated are quite diverse, and include individuals with congenital hearing loss of genetic origin (who are often children), those with acquired hearing loss due to cochlear nerve disruption from head trauma, those with NF2-associated tumors (typically vestibular schwannoma), those with non NF2-associated tumors, and those with severe cochlear ossification. Together, these make it difficult to assemble a large and homogeneous study cohort, which consequently tends to limit the statistical and inferential power of studies in this field.

A further complication for studies with ABI users is that it is often difficult for these patients to precisely communicate their hearing sensations, or to indicate their loudness perception. This makes the comparison between objective and perceptual thresholds challenging. This fact also illustrates the need for reliable objective measures that can be used to support audiological fitting in cases where behavioural responses cannot be reliably obtained.

In summary, the findings here indicate that cathodic-leading pulses may be the optimal method for stimulation with the ABI. For LEP measurements, the use of anodic pulses induces a latency shift which requires an artifact reduction method that takes this into account. Averaging cathodic- and anodic-leading pulses, as done in the alternating polarity method which implemented in the clinical software, may not be the most adequate method. The forward-masking paradigm used in this study would appear to be more promising.

## Methods

### Ethics, informed consent, and inclusion criteria

The collection of data for this study was approved by the Ethics Committee of the Hannover Medical School (9650_BO_S_2021). Informed consent was obtained from all participants and/or their legal guardians.

This study was designed and performed in accordance with the World Medical Association (Helsinki) declaration on ethical principles for medical research involving human participants, as well as the relevant local, national and international guidelines and regulations.

The criteria for inclusion in this study were users of MED-EL (Innsbruck, Austria) ABI devices of the i100 generation. No exclusion criteria were applied with regard to sex, age, or hearing loss etiology.

### Stimulation and recording of evoked responses

Electrical stimulation and response measurements were controlled using a MAX programming interface (MED-EL, Innsbruck, Austria). This was attached via USB to a computer where the research software was installed. A MAX Coil transmitting coil (MED-EL) was connected to the MAX programming interface and placed over the implant site (see Fig. 1b). Using the two-way telemetry functionality of the implant, this facilitated controlling the stimulus presentation and storing of the responses on the computer.

Stimulation and recording of LEPs was carried out using the MAESTRO clinical software applying the fine-grain recording paradigm. For each electrode contact, a stimulus strength sweep of the masker pulse was performed in a quasi-continuous manner from 0 qu (charge unit, 1 qu approximately equivalent to 1 nC) to the charge level corresponding to the maximum applicable loudness (MAL) level of the user for that electrode contact. Each stimulation strength increment was measured once.

The measurement of LEP is challenging because the stimulation produces an electrical stimulus artefact which is much larger in magnitude than the neural response. To mitigate the influence of the stimulus artefact, we chose used the revised forward-masking paradigm^21^. The forward-masking paradigm exploits the refractoriness properties of the neurons to cancel out the stimulation artefacts related to the masker and probe pulses by varying the inter-pulse interval (IPI) between the two pulses and the level of the probe pulse. The residual stimulus artifact related to the masker is most prominent in the two traces measured with the short IPI (traces E and F in Fig. 1d) and is, therefore, mitigated by the subtraction of the two traces from each other. The residual stimulus artifact related to the probe is the strongest in traces B and E (Fig. 1d), and only the neural response and the recording electrode artefact are thought to remain when the trace E is subtracted from trace B. Finally, the recording electrode artifact is then to be cancelled by subtracting the response of the fully recovered neuron to the zero-amplitude probe stimulus (trace C in Fig. 1d).

Following this paradigm, neural responses over a 1.7 ms duration were recorded at each charge unit level for traces B, E, C and F at a sampling rate of 1.2 MHz (Fig. 1c)^21^. Within a given stimulation sequence, the strength of the probe pulse for traces B and E was set as 90% of the strength of the masker pulse^21^, while for traces C and F a zero-strength probe pulse was used. The inter-pulse interval (IPI) is defined here as the duration from the offset of the masker pulse to the onset of the probe pulse. The inter-pulse intervals were chosen such that the smaller IPI would fall within the expected absolute refractory period of the neuron’s response function, and the larger IPI would allow full recovery before presentation of the probe. IPIs of 7.5 ms for traces B and C and 0.4 ms for traces E and F were used, following the work of Miller et al.^21^ and Westen et al.^34^ under the assumption that similar refractoriness and recovery properties would be observed in neurons of the CN as with those of the cochlear nerve.

Depending on the experimental condition, either anodic- or cathodic-leading pulses were used, as depicted in Fig. 2c. All probe and masker pulses were biphasic, charge-balanced, and used a 2.1 µs inter-phase gap. The phase duration depended on electrode contact impedance and the supply voltage-based compliance limit of the implant, which was derived by performing impedance field telemetry measurements prior to the LEP measurements. Impedance field telemetry was carried out in a similar fashion as in the clinical software. The pulse duration was chosen to ensure that the compliance level was not reached.

### LEP pre-processing, averaging, and artifact reduction

Before the final LEP responses could be obtained, the measured LEP responses were pre-processed and averaged across adjacent stimulus strengths. In the original fine-grained stimulation paradigm (as implemented in the clinical software), these two processing steps were separated. The neural responses were first filtered with a low-pass filter to remove interfering components (i.e., artifacts and noise), after which the noise level was further reduced by averaging over adjacent stimulus intensities. The rationale for this approach is that error introduced by averaging responses from different stimulus strengths is small or negligible when the step size is small^20^. Here, the two pre-processing steps were combined, and a 2D filter was applied for denoising in both domains simultaneously. To that end, the responses for B, E, C and F traces were each expressed as a 2D image with one axis representing time after stimulus onset and the other axis representing the stimulus strengths. Then, an anisotropic Gaussian kernel^35^ was used to smooth the 2D image. Standard deviations of 30 and 5 were chosen for the time and stimulus strength domains, respectively. This effectively corresponds to averaging over approximately ± 5 adjacent stimulus strengths, and filtering of the individual responses with a low-pass filter with a 3 dB cutoff frequency around 5.8 kHz. It should be noted that a similar 2D filtering approach was applied by Gärtner et al.^36^ for processing eCAPs measured with the fine-grain stimulation paradigm and was found to yield similar results as the traditional two-stepped processing. This 2D filtering was implemented using SciPy and NumPy in Python version 3.8^37,38^. After pre-processing the B, E, C, and F traces, the revised forward-masking artifact reduction was applied according to the method of Miller et al.^21^:

$$R = B - E - C + F,$$

Finally, the responses were down-sampled by a factor of ten, reducing the sampling rate effectively to 120 kHz. Figure 1c illustrates a plot of exemplary LEP responses for one participant measured at a single recording electrode.

The LEP responses were classified as clipped if any of the B, E, C or F traces were clipped. Clipped LEP responses were excluded from any subsequent analysis. In other words, responses that were not within the linear signal-processing range of the implant were excluded from the analysis.

### LEP threshold and amplitude growth function estimation

Since the peak minimum (N) of the LEP response is often cut off or non-existent (see Fig. 1c), the overall response amplitude was defined as the amplitude difference between P and the baseline, which was defined as the average amplitude within the last 425 µs of the response. Subsequently, a sigmoidal function $y\left( x \right) = y_{0} + \frac{A}{{1 + e^{{ - \frac{{\left( {x - x_{0} } \right)}}{D}}} }}$ was fitted to each amplitude growth function using the Levenberg–Marquardt algorithm^39^. Here, *x* is the stimulus strength of the probe pulse (nC), *y* is the amplitude of the LEP (µV), ${y}_{0}$ is the amplitude at baseline (i.e., the level of spontaneous activity without electrical stimulation, µV), *A* is the maximum observable response amplitude (µV), ${x}_{0}$ is the stimulus strength corresponding to the inflection point of the sigmoidal function (nC), and *D* is related to the dynamic range of the to-be-fitted function in qu.

By fitting these parameters, analytical estimates of the LEP threshold (defined as ${x}_{0}$*- D*) and the slope of the amplitude growth function (defined as *A/4D*) were obtained. The fitting of the sigmoidal function and estimation of LEP thresholds and amplitude growth function slopes was implemented using SciPy and NumPy in Python version 3.8^37,38^. The estimates for the LEP threshold, AGF slope and P latency were not considered for further analysis if the quality of the sigmoid function fitting (R^2^) was less than 0.8 or the estimated threshold value was negative.

### Perceptual thresholds

The perceptual threshold levels were obtained from the users’ routine audiological fitting sessions. These were obtained on the same date as the LEP measurements. The MCL and the hearing thresholds were acquired via behavioural testing. These records also noted which electrode contacts produced hearing sensations and which contacts produced non-auditory sensations or no sensation. To gain this information, individual electrodes were stimulated, and users were asked if they had a hearing impression. Along with this, they were presented with a visual illustration depicting scale ranging between quiet, medium and loud, which was used to indicate the intensity of their hearing percept. Users were also asked to report any side effects they experienced. In pediatric users who could not answer these questions, the levels were obtained by carefully observing whether the user reacted in any way to the stimulation. Stimulation was stopped immediately if the user showed any signs of discomfort. The lowest stimulation strength where a hearing impression was reported, or the lowest stimulation strength to which a reaction us without discomfort was observed was determined to be the perceptual threshold level.

### Statistical analysis

The first analysis used linear mixed-effects models to assess if and how the LEP thresholds and/or the LEP AGF slopes differed between the pulse shapes. For both the LEP threshold and the LEP AGF slope, a similar model was fitted to investigate the effects of pulse polarity (anodic-leading versus cathodic-leading) and electrode contact number (1–12) and their interaction as the dependent variables, with the participant ID being modeled as a random variable. That is, the following formula:

$${\varvec{y}} = X{\varvec{\beta}} + Z{\varvec{b}} + \varepsilon$$

was used to model the relationship between the observations *y* (LEP threshold or LEP AGF slope) and the fixed effects vector ***β***, with $X$ and *Z* being the design matrices for the fixed and random effects, respectively, and ${\varvec{b}}\sim N(0, {\varvec{D}})$ being the random effects vector describing the variance attributed to individual subjects and $\varepsilon \sim N(0, \boldsymbol{\Sigma })$ being the error vector^40^. ANOVA was then applied on the fitted models.

The second analysis was performed to investigate the relationships between the LEP thresholds obtained for anodic- and cathodic-leading pulses and the perceptual hearing thresholds. Electrodes that did not evoke auditory sensation were excluded from this analysis. Only electrodes where both an LEP threshold could be measured with the given pulse shape and information about the hearing threshold was available were included.

In total, 30 electrodes were excluded due to missing hearing threshold values (six for participant #01, six for participant #02, one for participant #06, six for participant #10, seven for participant #16 and four for participant #17). In addition, 17 electrodes were excluded from the analysis because of non-measurable LEP thresholds. These included 12 electrodes for anodic-leading pulses (seven for participant #04, three for participant #05, one for participant #6 and one for participant #09) and 5 electrodes for cathodic-leading pulses (three for participant #04, one for participant #06 and one for participant #09). Altogether, this resulted in 34 and 41 paired data points for anodic-leading and cathodic-leading pulses, respectively. Pearson correlation was used to estimate the relationships between the LEP thresholds and the perceptual thresholds.

The third analysis investigated variations in P latency between pulses with different leading polarities. P latencies were selected for inspection and found to reach more stable values with higher stimulation amplitudes, i.e. stimulation levels that were well above the threshold level. Therefore, the P latencies were first averaged across the last 10% of traces obtained at a given recording electrode during the LEP AGF measurement. The median P latency was calculated across the recording electrodes to obtain the P latency value for the given stimulating electrode contact for each participant. The P latencies for anodic- and cathodic-leading pulses were compared using paired t-tests.

## Conclusion

This study presents a new approach to recording LEPs from the CN using a fine-grained stimulation paradigm with a forward-masking artifact reduction scheme. The introduction of a baseline-dependent definition of the LEP amplitude can facilitate more consistent analysis of AGFs. The existing artifact reduction method implemented in MED-EL ABI systems is the alternating polarity scheme. In this study, we observed that the forward-masking paradigm seems to be more adequate, due to the latency shift between the cathodic- and anodic-leading pulse shapes. These findings suggest the use of cathodic-leading pulses in coding strategies for the ABI. The acquisition of LEPs may serve as a promising objective measurement to assist clinicians in device fitting.

## Supplementary Information

Supplementary Information 1.

Supplementary Information 2.

## Data Availability

The datasets generated during and/or analyzed during the current study are included in this published article and its Supplementary Information files.

## Abbreviations

ABI: Auditory brainstem implant
AC: Anodic-leading biphasic pulse
AGF: Amplitude growth function
CI: Cochlear implant
CN: Cochlear nucleus
CA: Cathodic-leading biphasic pulse
DCN: Dorsal cochlear nucleus
eCAP: Electrically evoked compound action potential
IPG: Inter-phase gap
IPI: Inter-pulse interval
MAL: Maximum applicable loudness
MCL: Most comfortable loudness level
NF2: Neurofibromatosis type 2
LEP: Local evoked potential
qu: Charge unit
VCN: Ventral cochlear nucleus
